# Analysis of a Vegetable Oil Performance in a Milling Process by MQL Lubrication

**DOI:** 10.3390/mi13081254

**Published:** 2022-08-04

**Authors:** Inês S. Afonso, José Pereira, António E. Ribeiro, Joana S. Amaral, Nuno Rodrigues, José R. Gomes, Rui Lima, João Ribeiro

**Affiliations:** 1 Instituto Politécnico de Bragança, Campus de Santa Apolónia, 5300-253 Bragança, Portugal; 2 Centro de Investigação de Montanha (CIMO), Instituto Politécnico de Bragança, Campus de Santa Apolónia, 5300-253 Bragança, Portugal; 3 Laboratório Associado para a Sustentabilidade e Tecnologia em Regiões de Montanha (SusTEC), Instituto Politécnico de Bragança, Campus de Santa Apolónia, 5300-253 Bragança, Portugal; 4 CMEMS–UMinho, Universidade do Minho, 4800-058 Guimarães, Portugal; 5 LABBELS–Associate Laboratory, 4800-058 Guimarães, Portugal; 6 MEtRICs, Mechanical Engineering Department, University of Minho, Campus de Azurém, 4800-058 Guimarães, Portugal; 7 CEFT, Faculdade de Engenharia da Universidade do Porto (FEUP), Rua Roberto Frias, 4200-465 Porto, Portugal; 8 ALiCE, Faculty of Engineering, University of Porto, 4200-465 Porto, Portugal

**Keywords:** cutting fluid, vegetable oil, milling process, MQL lubrication, micro and nanofluidics

## Abstract

In this work, we carried out a comparison between the dry machining of an aluminum block with conventional cutting oil and a block with vegetable oil. The two oils had different flow rates. Using the Taguchi method, it was possible to determine the matrices for optimizing the best parameters for each group of tests. Then, we studied the utility of using vegetable oil as a cutting lubricant. We found that the vegetable oil studied in this work had good properties in terms of reducing cutting temperatures but was less effective than conventional cutting oil in reducing the surface roughness of the machined part. Tribological tests were carried out to understand the influence of the selected lubricants in reducing friction and wear. After the sliding experiments, which were performed without lubrication in the presence of the same lubricants that were used in the machining tests and in the presence of distilled water, we concluded that vegetable oil has satisfactory lubricating properties that are similar to those of the conventional cutting fluid, indicating a potential for consideration as an effective alternative to the conventional cutting fluid, with economic, environmental, and health advantages.

## 1. Introduction

Milling is one of the most common machining processes [1], at both the macro- and the microscale levels [2,3,4,5]. In the milling process, extreme heat generation in the tool–workpiece interface influences the quality of the products and tool life considerably. It increases the cutting temperature and lowers the surface quality. Furthermore, the friction that is induced in machining processes has negative impacts on both the workpieces and the tools [6,7]. Consequently, lubrication and cooling are important in the milling process.

Cutting fluids play an important role in milling processes because they offer adequate conditions for material cutting by improving the cooling, reducing the friction, and removing the produced chips [8]. In addition, the machining parameters that are necessary to obtain quality products with low tool wear, in an economically viable time, must be taken into account [9]. To manufacture a particular part, there is a machining process that is most suitable in providing the greatest quality for the lowest machining time and energy consumption. The selection of the process depends on the objective; in machining, the selection of the process is related to the part’s material and its geometry, as well as to the machines and the tools that are available. Based on the aim of the machining aim and the selection of cutting tool, there are different combinations of parameters to obtain different results in terms of the quality of the machined surface and tool wear, such as spindle speed, feed rate, and axial or radial depth of cut. [10,11]. The various cutting parameter combinations result in variations in surface roughness and tool life. It is very difficult to define the best combination of parameters that will provide the lowest surface roughness and the maximum tool life. To overcome these difficulties, many researchers have tried different approaches using mathematical algorithms for use in experimental tests to predict the surface roughness resulting from machining [12] and to optimize solutions [13,14,15].

As mentioned, the machining process demands a large usage of energy, with most of the consumed power being converted into heat near the cutting edge so that the tool and chip interface can reach very high temperatures (up to 1100 °C). However, such high temperatures can result in poor surface finishes [14], residual stresses [15], cracks [16], and, in some cases, a reduction in tool life [17]. To achieve the best machining performance with reduced temperatures during machining, several studies have been performed to develop new cutting fluids that can reduce the friction coefficient and improve cooling performance [18]. Presently, metalworking fluids (MWFs) are part of a large family of lubricants that are available because of the great number of different metal-cutting processes. Most MWFs are petroleum-based. With technological progress, industries require the development of fluids that have specific functions for machining different kinds of materials. Among the MWFs, there are three main groups: straight or neat oils, emulsions, and solutions. Each type of MWF can be a mineral fluid or a vegetable oil. For each machining process, it is necessary to select the most suitable fluid in order to achieve the best performance, which includes considering the different chemical additives that are used to formulate MWFs that perform multiple functions. These functions include emulsification, corrosion inhibition, lubrication, microbial control, pH buffering, coupling, defoaming, dispersing, and wetting [19]. An aggressive manufacturing environment promotes the development of MWFs that are favourable for microorganism proliferation, temperature oscillations, humidity variations, and usage in factory environments [20]. 

Vegetable oils are mainly composed of triacylglycerol molecules that have two main parts, glycerol and fatty acids [21]. Compared with mineral oils, vegetable oils have superior lubrication properties due to their molecular composition and their chemical structure [21]. In addition, they have a relatively high viscosity index compared with mineral-based oils, enabling them to operate in a wider temperature range—a desirable characteristic [22]. The properties of vegetable oils are directly influenced by the composition of fatty acids and the degree of saturation. As a result, the efficacy and performance of vegetable oils allow them to be used as lubricants [21]. Compared with mineral-based lubricants, vegetable oils generally have several advantages, such as high flash points, high viscosity indices, higher lubricities, low evaporative losses, and good metal adherence. However, these oils have some drawbacks, including low thermal stability, poor oxidative stability, poor low temperature properties, and poor corrosion protection [22]. To overcome these limitations, researchers have applied both micro- and nanotechnology in developing a new generation of MWFs, known as “nanofluids” [23,24,25,26,27,28,29]. The nanofluids are based on biological fluids, to which nanoparticles are added [29]. Many researchers are involved in developing biological cutting fluids from vegetable oils with nanoadditives [29], as the thermal limitations found in vegetable oils can be improved by the addition of nanoparticles with superior heat transfer capabilities [28].

Different vegetable oils, such as canola, rapeseed, and coconut oil, have been studied as alternatives to mineral cutting oil for use in machining processes [30,31,32]. The results of these studies prove that vegetables oils are a viable alternative, as they have interesting natural properties, such as adequate lubrication, high viscosity indices, high flashpoints, and environmentally friendliness. However, they have low oxidation stability at high temperatures [33,34,35]. Olive oil is one of the vegetable oils that is most commonly produced in Mediterranean countries. In 2021/2022, Portugal had the highest growth rate among European countries in the production of olive oil, producing 206,000 tons of olive oil—the greatest production ever achieved by this country [35].

Traditionally, the cutting fluids used in machining processes are applied by a flood flow system; however, this process requires an enormous quantity of fluid, which is expensive, and which can have negative impacts, such as water and groundwater contamination, air pollution, and soil contamination. To avoid the risks of cutting fluids and to achieve cleaner and healthier production, MQLs (minimum quantity lubrications) have been created. As the name indicates, this lubrication system uses a very small amount of cutting fluid, between 6 mL/h and 100 mL/h [36]. Therefore, MQLs can provide an alternative to flood machining and dry machining, using compressed air at a high speed (100 m/s). A small amount of cutting fluid is injected into the cutting zone in the form of ultra-fine drops [37], a process that has been accepted as a clean form of lubrication in the context of sustainable production, based on environmental preservation in compliance with ISO 14,000 [36].

In this work, a mixture of water with olive oil was used as cutting fluid in a milling process. The cutting fluid was pre-prepared to obtain a nanoemulsion of olive oil–water surfactant [38,39,40], which was applied in the process of an MQL system. Its performance was characterized by measuring the temperature of metal chips removed from workpiece and by the surface roughness measured after a face-milling operation. To evaluate the influence of the milling parameters and the cutting fluid, designed experiments based on the Taguchi method were carried out. The Taguchi method was used to define the orthogonal array of the experimental tests within which it was possible to combine several parameters to obtain an optimal combination for a given factor that was being studied, such as surface roughness. The Taguchi method is also associated with statistical tools, such as ANOVA analysis, that include a degree of uncertainty in the practical results in order to understand future results. In addition to the experimental machining tests, a tribological characterization (friction coefficient and wear analysis) was also performed, in which the biological cutting fluid that was used was compared with a commercial fluid.

## 2. Materials and Methods

**Figure 1:**
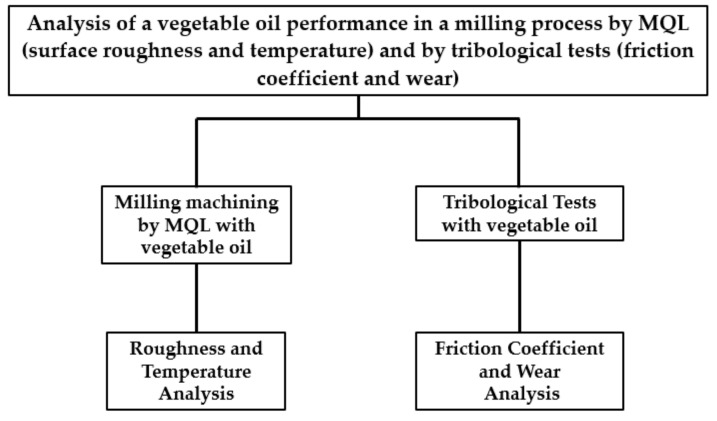
Diagram of the performed analysis.

For this study, two major analyses were performed, machining (milling) tests and tribological tests. In the machining tests, the MQL system of lubrication was used, with the control parameters of surface roughness and chips’ temperatures. In the tribological tests, the friction coefficient and the wear on a stainless-steel disk after sliding against an alumina ball were measured for different conditions, i.e., dry sliding in the presence of a commercial cutting fluid and in the presence of a vegetable fluid. Figure 1 shows a diagram of the tests.

### 2.1. Materials and Experimental Design

In the first part of this work, the experimental machining tests were performed to compare the surface roughness and the milling temperatures, according to the type of lubricant used and the flow rate of each lubricant. The goal was to determine if vegetable oil has the potential to be used as a cutting fluid, when compared with the commercial cutting oil Balis MAFCOOL 51 (Balislub, Gondomar, Portugal).

Five groups of machining tests were carried out (Table 1), in which the differences were the type of lubricant and the flow rate.

All five groups used the same Taguchi array, as shown in Table 2.

The parameters used in the Taguchi array were the spindle speed (n), the feed rate (Vf), and the axial depth (ae). The chosen levels were in the range of the levels defined by the tool manufacturer, as shown in Table 3.

By dividing the milling tests into five groups, it was possible, using the signal-to-noise (S/Ns) ratio and the ANOVA test, to determine the optimal combination of parameters that had the lowest temperatures and surface roughness in each group, with different lubricants.

The next part of this study consisted of tribological tests that were performed in the presence of the conventional cutting fluid and the vegetal oil in order to compare the friction results in both sliding conditions and to understand how the friction coefficient decreased in comparison with the dry sliding condition.

Two different olive oils were used, one produced from a traditional milling process (Vegetable oil 1, Lamalonga, Macedo de Cavaleiros, Portugal) and the other from a three-stage olive oil extraction process (Vegetable oil 2, Arcas, Macedo de Cavaleiros, Portugal). Both oils were obtained from the northeast Portuguese village of Macedo de Cavaleiros. To guarantee the adequate emulsion and stability of the water and olive oil mixture, non-ionic surfactants were added. The preparation of this fluid was based on the work of Polychniatou and Tzia [38]. The surfactant used was polyoxyethylene sorbitan monopalmitate (Tween 60), acquired from Merck (Belize). The olive oil was previously filtered by filter paper of grade 1 (Whatman^®^, Merck KGaA, Darmstadt, Germany). The ratio, in weight, was 10% olive oil, 2% surfactant, and 88% water. Accurate quantities of surfactant and oil were mixed (final mixture of 100 g) using a high-speed homogenizer (FSH-2B, Lab Disperser Emulsifier, MXBAOHENG) at 10,000 rpm, and then the necessary quantity of water was added in drops to form a w/o nanoemulsion. The homogenization was set to 40 °C, as recommended by Polychniatou and Tzia. During the homogenization process, care was taken to avoid the formation of air bubbles.

### 2.2. Machining Experimental Tests

The experimental machining tests were performed by the milling operation on an aluminum alloy (AL6061) block (150 × 100 × 50 mm^3^). This material was chosen because of its good machining properties, such as high thermal conductivity, corrosion resistance, and low density.

The milling machine was an MRF model FU145, (Spain) and the experimental tests were performed with a face-milling tool, 100 mm in diameter, and the cutting inserts had the reference TPMN 220412 (LAZA CNC, Shenzhen, China).

**Figure 2:**
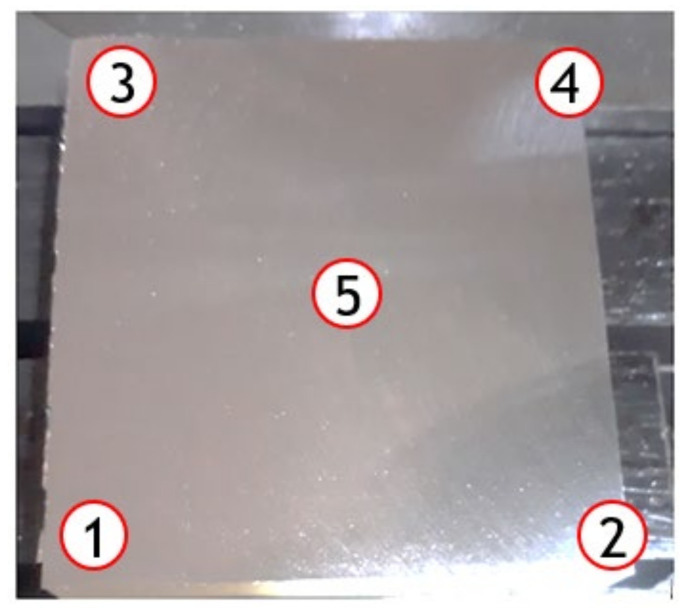
Details of surface roughness measurement points on aluminum alloy block.

The surface roughness was measured using a portable surface roughness tester (Surftest SJ 301, Mitutoyo, Tokyo, Japan) at five different locations on the block of aluminum (Figure 2). The arithmetic average of roughness profile (R_a_) was used as it is one of the most widespread surface roughness parameters employed in the industry and in the scientific community.

**Figure 3:**
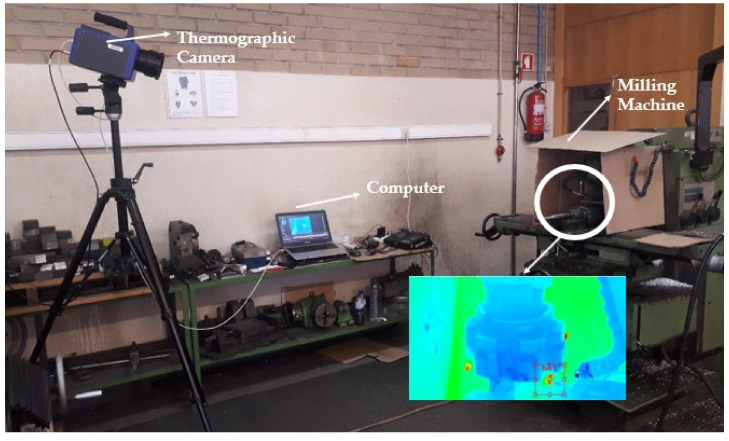
Test setup for the chip temperature analysis via ResearchIR Max software.

The temperatures of the metal chips were measured using the thermographic cam-era FLIR SC7000 (Teledyne Flir, Wilsonville, OR, USA) and 20 values of the temperatures of the chips were later analyzed with ResearchIR Max software. For these values, the arithmetic mean was used to obtain the average chip temperature of each test. The setup of the test is shown in Figure 3.

To spray the cutting fluids, an atomizer (ZIGUA, model YS-BPV-3000, Hoenyzy, Wenzhou, China) was used which is a mixer block that regulates airflow and lubricant flow. The equipment consisted of a regulation block, a flexible tube, an outlet nozzle, two flow regulators, and two inlets for compressed air and cutting fluid. Through the adjustment block, it was possible to regulate the airflow and the cutting fluid. This adjustment was obtained using a chamfered screw and a 2.5 mm hole inside the block. Thus, by moving the screw toward or away from the orifice, the area of the section of the fluid passage changed, varying the flow rate. With each complete rotation (360 degrees), the screw moved 0.75 mm, determining the section area from 45° to 45°. Only Vegetable oil 2 was used to perform these experimental tests because the tribological tests verified that its friction coefficient was slightly lower than that of Vegetable oil 1. The flow rate was determined using a calibrated syringe and a chronometer, changing the previous atomizer parameters until 50 mL/h and 100 mL/h values were obtained.

### 2.3. Tribological Tests

**Figure 4:**
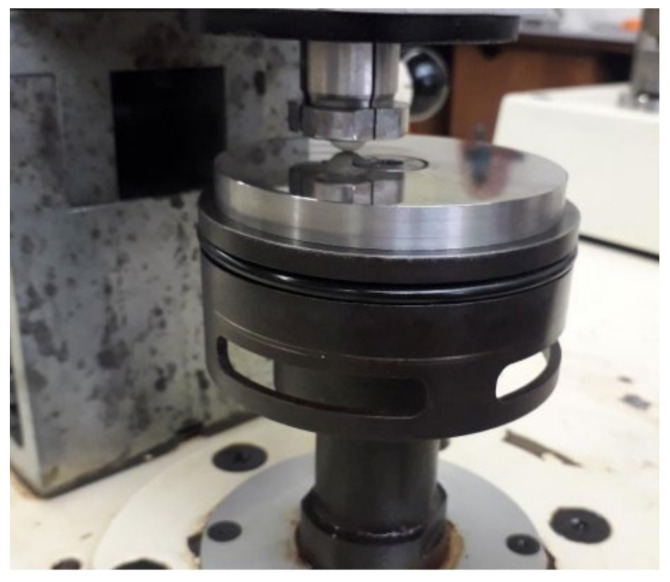
Sketch of the pin-on-disk. The dimensions of the pin were 32 mm (l) × 10 mm (dia) and dimensions of the disk were 8 mm (l) × 163 mm (dia).

The tribological tests were performed with a PLINT TE67 tribometer (Phoenix Tribology Ltd., Berkshire, England), which is a universal device intended for friction and sliding wear tests and which facilitates several test geometries, such as pin-on-disc, which was the one used in this study. The tests were performed using an alumina ball against a stainless steel 316L rotating disc, as shown in Figure 4. The sliding speed and the normal applied load were kept constant for all tests, assuming the values of 5 N and 0.1 m/s, respectively. The tests were performed in the absence of any fluid (dry sliding) and in presence of different fluids, i.e., distilled water, cutting oil Balis MAFCOOL 51, and vegetable oil. For each test, the friction coefficient evolution during sliding was recorded and the average friction coefficient in the steady-state regime was evaluated. The wear of the ceramic ball was determined from the weight loss, which was measured using a microbalance with an accuracy of 10 μg.

The wear results were expressed in terms of the specific wear rate, calculated according to Equation (1):

(1) $$K=\frac{V}{W\times x}$$

where K is the specific wear rate [mm^3^/N.m], V is the wear volume [mm^3^], *W* is the normal load [N], and x is the sliding distance [m].

## 3. Results and Discussion

### 3.1. Machining Performance

For each machining test, the average of the five results on the surface roughness of the workpiece and the twenty results of the chip temperatures were determined. After that, the average of the surface roughness was calculated for each group of lubrification. These results are shown in Table 4.

The cutting test condition corresponding to the lowest average surface roughness was Balis MAFCOOL 51, with a flow rate of 100 mL/h ± 5 mL/h, and the dry test obtained the highest value of average surface roughness. In all of the test groups, test number seven had the lowest surface roughness value.

For the average chip temperature results, the Balis MAFCOOL 51 (BMAF 51) test with a flow rate of 100 mL/h ± 5 mL/h was characterized by the lowest average temperature.

#### 3.1.1. Optimal Combination of Parameters

In the machining process, one of the most important goals is to minimize the surface roughness of the part and the machining temperature (which can be evaluated by the chip’s temperature). The most appropriate control factor is the smallest sign-to-noise ratio (S/Ns), which can be defined by Equation (2).

(2) $$\frac{S}{N_{s}}=-10\times \log\left(\frac{1}{n}\sum_{i=1}^{n}y_{i}^{2}\right)$$

where n is the number of observations and y is the observed data.

Table 5 shows the S/Ns values for the average surface roughness and the average chip temperature, which were obtained in the machining tests performed without lubrication (dry). In the other machining tests with lubrification, the behaviour of the S/Ns values followed the same pattern.

It was also possible to calculate the S/Ns ratio for each parameter relative to the average surface roughness and the chip temperature, as shown in the Table 6.

**Figure 5:**
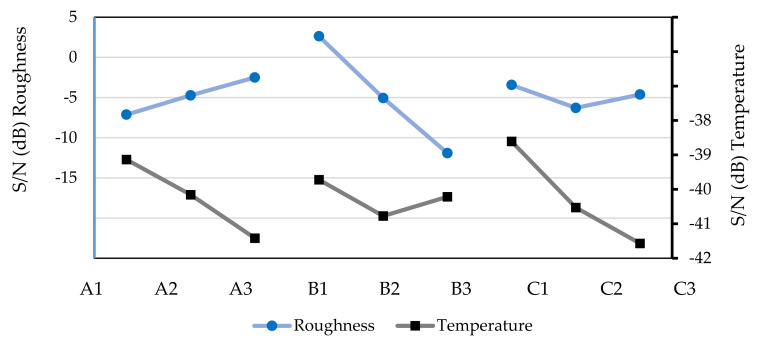
S/Ns to surface roughness of the workpiece and chip temperatures based on the machining tests without lubrication (dry).

Using the data shown in Table 6, the graph shown in Figure 5 was created, in which it was possible to define the optimal combination for obtaining lower chip temperatures and lower surface roughness of the workpiece.

After analyzing Figure 5, to obtain a low surface roughness of the workpiece in the dry machining test, the optimal parameters were A3B1C1, i.e., a spindle speed of 500 rpm, a feed rate of 20 mm/min, and an axial depth of 0.3 mm. The optimal parameters for a low chip temperature must be A1B1C1; that is, a spindle speed of 195 rpm, a feed rate of 20 mm/min, and an axial depth of 0.3 mm.

**Figure 6:**
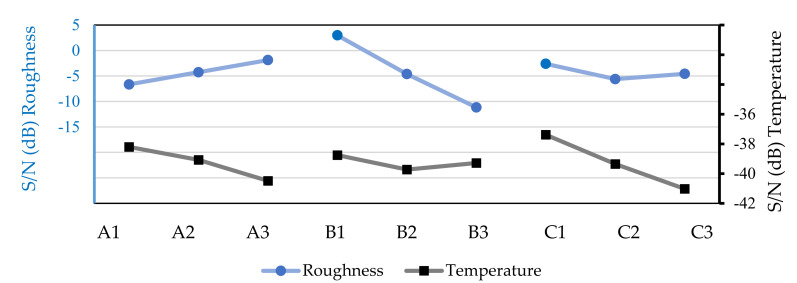
S/N values for surface roughness of the workpiece and chip temperatures based on the machining tests with Balis Mafcool 51 (50 mL/h).

**Figure 7:**
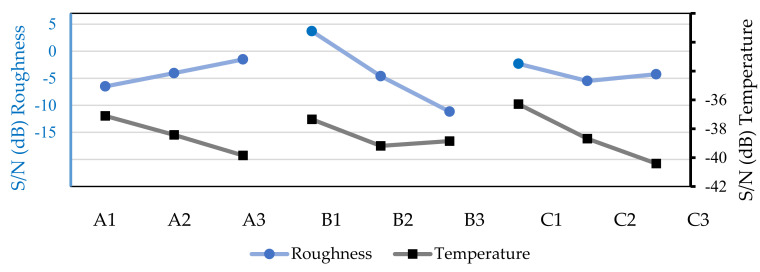
S/N values for surface roughness of the workpiece and chip temperatures based on the machining tests with Balis Mafcool 51 (100 mL/h).

**Figure 8:**
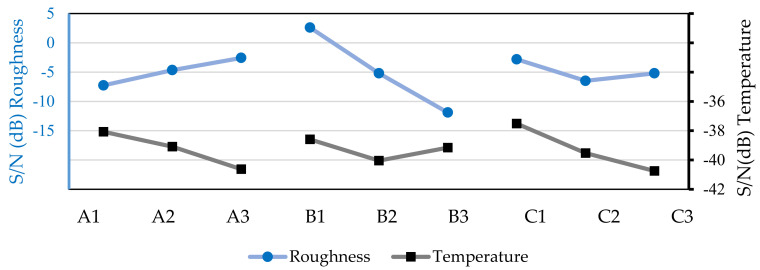
S/N values for surface roughness of the workpiece and chip temperatures based on the machining tests with vegetable oil (50 mL/h).

**Figure 9:**
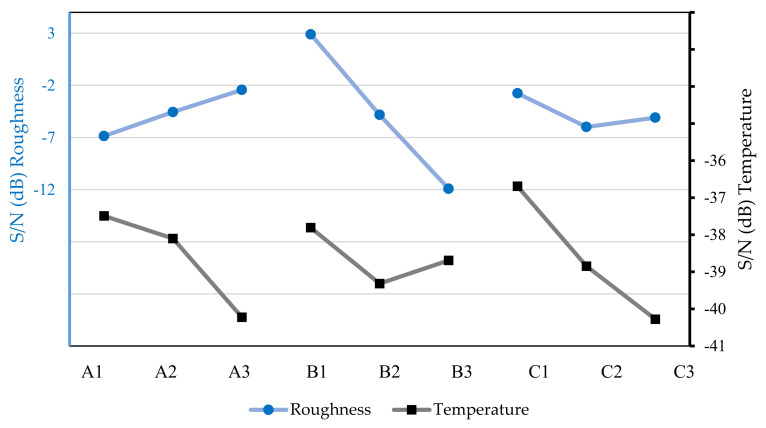
S/N values for surface roughness of the workpiece and chip temperatures based on the machining tests with vegetable oil (100 mL/h).

In the same way, it was possible to obtain the optimal combination for the other lubricant conditions, as shown in Figure 6, Figure 7, Figure 8 and Figure 9.

Table 7 shows the values of R_a_ and the chip temperatures measured for the machining tests using the MQL lubrication with Balis Mafcool 51 (50 mL/h), and the S/Ns values determined from the experimental data.

Figure 6 shows the surface roughness of the workpiece and the chip temperature values based on the machining tests with the industrial lubricant, with a flow rate of 50 mL/h, it can be seen that to choose an optimal combination for low surface roughness, the combination A3B1C1 must be used, i.e., a rotation of 500 rpm, a feed speed of 20 mm/min, and penetration of 0.3 mm. To obtain lower cutting temperature values, the combination A1B1C1 must be used; i.e., a spindle speed of 195 rpm, a feed rate of 20 mm/min, and an axial depth of 0.3 mm. These combinations are the same for the group of tests without lubrication, as shown in Figure 5.

For the next group of machining tests, the surface roughness of the workpiece and the chip temperature values on the machining tests, with the industrial lubricant with a flow rate of 100 mL/h, are shown in Table 8 and analyzed in Figure 7. They indicate the same optimal combinations for these two parameters; i.e., for surface roughness, the combination A3B1C1 was used, corresponding to a spindle speed of 500 rpm, a feed speed of 20 mm/min, and an axial depth of 0.3 mm, and to achieve low cutting temperatures, the optimal combination obtained was A1B1C1, with a spindle speed of 195 rpm, a feed speed of 20 mm/min, and an axial depth of 0.3 mm.

Table 9 shows the values of Ra obtained for the workpiece and the chip temperatures measured for the machining tests using the MQL lubrication with vegetable oil and a flow rate of 50 mL/h, as well as the S/Ns values determined from the experimental data.

Analyzing Figure 8, it is possible to verify that with the use of vegetable oil, the S/N values for surface roughness and the chip temperatures follow the same trend as in the previous trial groups. The increase in spindle speed favors a good surface finish (lower surface roughness) but increases the cutting temperature. The increase in feed speed increases surface roughness, and the increasing axial depth causes an increase in chip temperature. Based on Figure 8, it is also possible to state that the optimal combinations for low surface roughness and low chip temperatures are the same as those of the previous machining tests. For low surface roughness, the combination A3B1C1 must be used, corresponding to a spindle speed of 500 rpm, a feed speed 20 mm/min, and an axial depth of 0.3 mm. On the other hand, for low chip temperatures, the best combination is A1B1C1, with a spindle speed of 195 rpm, a feed speed of 20 mm/min, and an axial depth of 0.3 mm.

Table 10 shows the experimental results for the machining tests using vegetable oil, with the flow rate of 100 mL/h.

Based on Figure 9, it can be stated that, as in tests previously mentioned, increasing the cutting speed favors an increase in the signal-to-noise ratio for the surface roughness, which results in a better surface finish of the workpiece. This increase in spindle speed has a deleterious impact on chip temperatures, which will increase. The additional increase in speed does not favor the surface finish; i.e., with the decrease in the signal/noise ratio, an increase in the surface roughness of the machined part is expected. Regarding axial depth, the greater the penetration, the greater the value of the cutting temperature.

As indicated previously for the conditions of the machining tests, the optimal combinations for the lowest surface roughness of the part and the lowest chip temperatures are A3B1C1, corresponding to a spindle speed of 500 rpm, a feed speed of 20 mm/min, and an axial depth of 0.3 mm, and A1B1C1, with a spindle speed of 195 rpm, a feed speed 20 mm/min, and an axial depth of 0.3.

#### 3.1.2. ANOVA Analysis

To assess the effectiveness of each parameter, an analysis of variance was performed. The level of influence of each control factor on a specific response was determined. In this case, the ANOVA analysis was performed for the surface roughness and the cutting temperature. The results of variance for the degrees of freedom (Df), the sum of squares (Sq), the mean of squares (Md), and their interactions in the dry machining test are shown in Table 11.

Based on Table 11, it is possible to conclude that the parameter with the greatest influence on the increase in chip temperatures in dry machining is the axial depth, followed by the spindle speed, with contributions of 56.99% and 33.10%, respectively. The parameter with the least influence on the increase in chip temperatures is the feed rate, with a contribution of only 7%. Thus, to reduce machining temperatures, lower axial depth and lower spindle speed should be used.

Regarding the increase in surface roughness of the workpiece, the parameter with the greatest contribution was the feed rate, followed by the spindle speed, with contributions of 84.92% and 8.60%, respectively. The Axial depth had a contribution of only 3.30%. In all of the other machining test groups, the same behaviour was maintained.

Table 12 shows the relevant values, with lubrication conditions by Balis Mafcool 51, with a flow rate of 50 mL/h.

Based on Table 12, it is possible to affirm that with the application of the commercial cutting fluid with a flow rate of 50 mL/h, the parameter with the greatest contribution to the increase in machining temperature was the axial depth, with a contribution of 66.42%. The spindle speed was next, with a contribution of 26.65%; the feed rate’s contribution was only 4.67%. Regarding the surface roughness of the workpiece, the feed rate was the parameter with the highest contribution, 83.22%, followed by the spindle speed (9.50%) and the axial depth (3.84%).

Table 13 shows the relevant values with the same lubricant, but with a flow rate of 100 mL/h.

Based on Table 13, the same behaviour as in the previous tables was verified. The axial depth was the parameter with the greatest contribution to the machining temperature increase, followed by the spindle speed and, finally, by the feed rate (56.33%, 24.78%, and 12.65%, respectively). For surface roughness, the feed rate was the parameter with the largest contribution (83.72%), followed by the spindle speed (9.39%) and the axial depth (3.88%).

Table 14 shows the relevant values for the results of the application of vegetable oil with a flow of 50 mL/h.

Based on Table 14, it was verified, once again, that the contribution of the cut-off parameters in the two responses that were studied followed the same trend. For the machining temperatures, the axial depth was the parameter with the highest contribution (50.46%), followed by the spindle speed (31.30%) and the feed rate (10.07%). For surface roughness, the feed rate was the parameter with the greatest contribution (82.02%), followed by the spindle speed and the axial depth (8.59% and 5.40%, respectively).

Table 15 shows the relevant values with the same lubricant as before, but with a flow rate of 100 mL/h.

Based on Table 15, it can be seen, once again, that the axial depth is the parameter with the greatest contribution to the increase in machining temperature (52.85%), and the feed rate is the parameter with the greatest contribution to the increase in surface roughness (83.75%). For the cutting temperature, the spindle speed stands out, with a contribution of 33.46%, followed by the feed rate with 9.29%. In terms of surface roughness, the spindle speed had a contribution of 7.49%, followed by the axial depth with 4.23%.

#### 3.1.3. Comparison of Lubricating Fluids

One of the main objectives of this study was to understand whether vegetable oil has the potential to be used as a cutting fluid. To compare the two fluids used (industrial cutting fluid Balis MAFCOOL 51 and vegetable oil), and to determine whether vegetable oil is viable as a machining lubricant, a parallel analysis was performed for the two fluids, focusing on the average surface roughness of the workpiece and the chip temperatures of each lubrication group.

Table 4 shows the results of average surface roughness of the workpiece and chip temperature values obtained in each machining test and for each test group with a different lubricant. It was possible to create a graphic illustration that shows the average chip temperature and the surface roughness obtained in each group, for each lubricant and flow rate.

**Figure 10:**
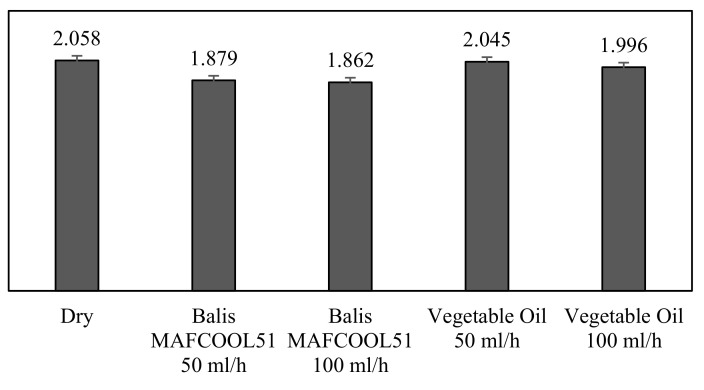
Average surface roughness of the workpiece in each group of machining tests; units in µm.

Figure 10 shows the mean of the average roughness of the workpiece obtained in each machining test group, with the variations in the cutting fluid and the flow rate.

Figure 10 shows that the average surface roughness of the workpiece from the dry machining test group corresponds to the highest value, R_a_ = 2.058 μm. Comparing the two fluids, it appears that for the same flow rates (50 and 100 mL/h), the conventional cutting lubricant (Balis MAFCOOL 51) always achieved a better contribution than vegetable oil. A flow rate of 50 mL/h conventional cutting fluid provided better results than vegetable oil with a flow rate of 100 mL/h.

The most efficient lubricant was the conventional cutting lubricant with a flow rate of 100 mL/h. Next was the same lubricant with a flow rate of 50 mL/h, followed by vegetable oil with flow rates of 100 mL/h and 50 mL/h.

**Figure 11:**
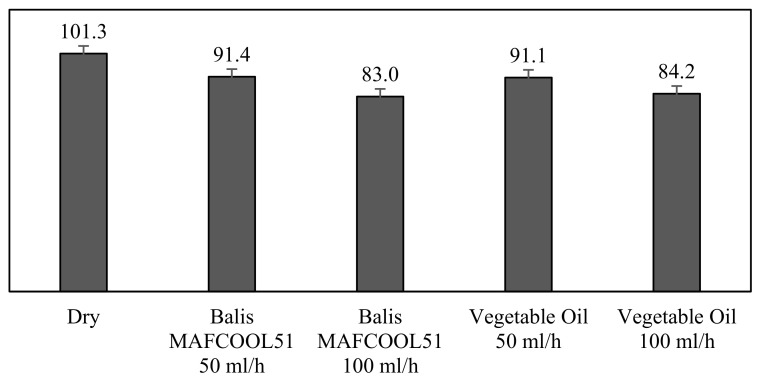
Average chip temperatures [(C) in each group of machining tests.

Figure 11 shows the mean chip temperature values for each machining test group, with the variations in the cutting fluid and the flow rate.

Figure 11 shows that the worst result was obtained with the dry machining test, with an average tip temperature of 101.3 °C. When comparing the lubricants used with a flow rate of 50 mL/h, the two fluids allowed a temperature reduction to around 91.4 °C.

By increasing the flow rate to 100 mL/h on the two fluids, we found that the conventional cutting fluid became only slightly more effective in reducing the machining temperature. The conventional cutting oil achieved a reduction to 83.0 °C, whereas a reduction to 84.2 °C was attained with the application of vegetable oil.

Although the viscosity of the mixture of olive oil and water that was used in this work has not yet been tested, it was assumed that it is similar to that of olive oil, which decreases with temperature reductions [41]. With this behaviour, vegetable oils had a higher wettability during temperature reductions, which meant that the tested vegetable oil mixture would be a good cutting fluid.

It can be concluded that vegetable oils had a greater impact on machining temperature reduction than on the surface roughness of the part. However, the cutting fluid was efficient in terms of the study’s two objectives. With the increase in the flow rate, there were improvements in its effectiveness.

### 3.2. Tribological Tests

#### 3.2.1. Friction Coefficient

**Figure 12:**
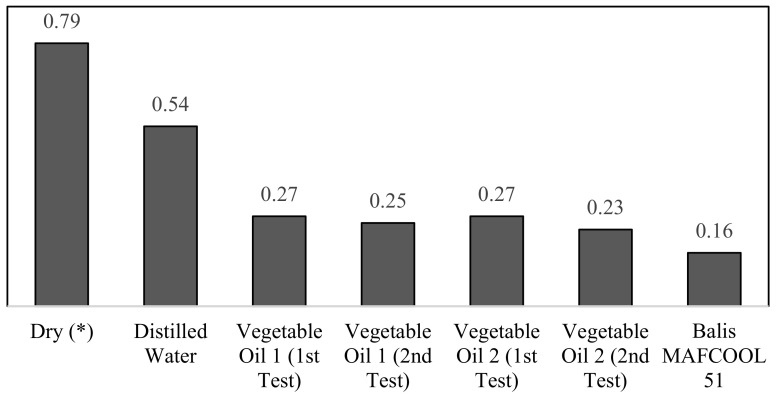
Friction coefficient values in steady-state regime for sliding contacts between an alumina ball and 316L stainless steel for different lubricant conditions (W = 5 N; v = 0.5 m/s).

The friction coefficient results, all relative to the alumina/stainless steel 316L sliding pair for the different lubrication conditions that were considered, are shown in Figure 12. Seven tribological tests were performed: without lubrication (dry), with Balis MAFCOOL 51 cutting oil, with Vegetable oil 1 (two tests), with Vegetable oil 2 (two tests), and with distilled water.

**Figure 13:**
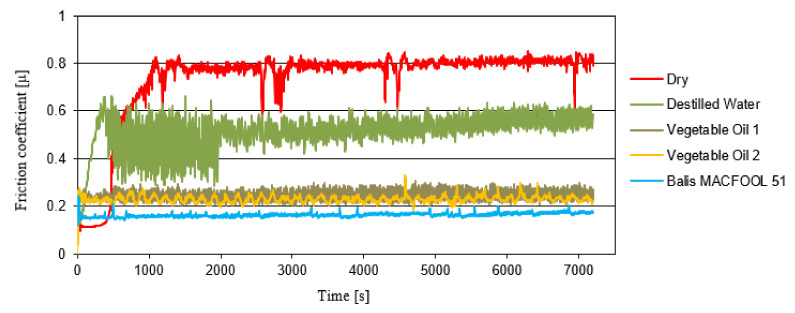
Comparison of the tribology tests of the evolution of the friction coefficient during sliding (alumina ball against stainless steel; W = 5 N; v = 0.5 m/s).

Each sliding test was carried out for 7200 s and the friction coefficient was continuously recorded. Figure 13 shows the friction coefficient evolution during sliding for the different test conditions that were considered (dry sliding and distilled water), the average of the tests performed with Vegetable oils 1 and 2, and the average with the Balis MACFOOL 51 cutting oil.

Based on Figure 12 and Figure 13, it is visible that, as expected, the dry sliding condition resulted in the highest friction coefficient (0.79), followed by the sliding test in presence of distilled water with an average friction coefficient in steady-state regime of 0.54. The sliding conditions in presence of Vegetable Oil 1 and Vegetable Oil 2 were characterized by a similar friction behaviour in both cases, with the former having an average friction coefficient of 0.26 and the last an average friction coefficient of 0.25. The industrial cutting fluid, Balis MAFCOOL 51, presented the lowest average coefficient value, 0.16, what is an expected result.

In terms of stability of the friction coefficient values during sliding, both the vegetables oils and the industrial cutting fluid showed relatively smooth friction coefficient evolution curves when compared to the sliding contacts in presence of distilled water or under dry condition.

#### 3.2.2. Wear Analysis

Wear analysis was determined on the basis of the results obtained in the tribological tests for the alumina ball, as shown in Table 16.

The results showed that the dry and the distilled water sliding tests could produce measurable wear on the alumina ball, of an order of magnitude higher under dry sliding than in presence of distilled water. However, no measurable wear was obtained when sliding occurred in the presence of vegetable oils or in the presence of the industrial cutting fluid, indicating the potential of te vegetable oils as a cutting fluid.

## 4. Conclusions

In this study, the possibility of using a vegetable oil as a cutting fluid in machining processes was evaluated. With the use of five different lubrication groups, we verified that the combination of ideal machining parameters remained unchanged, independent of the type of lubrication used.

Through ANOVA analysis, we concluded that the parameter with the greatest influence on the increase in machining temperatures was the axial depth, followed by the spindle speed. The parameter with the greatest contribution to the increase in surface roughness of the workpiece was the feed rate, followed by the spindle speed.

Our analysis of the surface roughness and the machining temperature for each l lubrication group showed that the use of the MQL technique with the conventional cutting fluid, with a flow rate of 50 mL/h, was a possible alternative to dry lubrication because it reduces the surface roughness and chip temperatures. With the increase in the flow rate to 100 mL/h, the surface roughness reduction was not significant (compared with the conventional cutting fluid with a flow rate of 50 mL/h), although there was a decrease in the machining temperature.

When vegetable oil was applied with both flows, the surface roughness remained very close to that of the dry condition, indicating that vegetable oil has little lubrication effect. On the other hand, our analysis of the machining temperatures with the application of vegetable oil indicated that the temperatures remained comparable to those obtained with the conventional cutting fluid, demonstrating that vegetable oils have high cooling power in machining processes.

We verified that the type of lubrication does not influence the choice of the ideal machining parameters, because the optimal combination for the different lubrication groups remained the same. Vegetable oils have great potential for reducing cutting temperatures. However, although vegetable oils allowed a reduction in the surface roughness of the workpiece, compared with dry machining, the obtained results with vegetable oils were slightly below the results obtained with the conventional cutting oil.

On the tribological tests, the two different vegetable oils did not show a significant difference in the friction coefficient values (0.26 and 0.25). Although those results were characterized by higher friction values than the friction coefficient obtained for sliding in the presence of the industrial cutting fluid, Balis MAFCOOL 51 (0.16), they can be considered satisfactory, indicating that vegetable oils are a good alternative to the industrial cutting fluid. The wear of the alumina ball was not measurable for sliding in the presence of vegetable oils; and the wear in the presence of the cutting fluid reinforces the conclusion that vegetable oils have potential as cutting fluids.

Finally, for the two vegetable oils that were analyzed, consisting of a mixture of water and olive oil, comparable results were obtained in both milling and tribological tests, indicating that they have high potential for use as a lubricant in milling operations as an effective alternative to the cutting fluid that was analyzed, Balis MAFCOOL 51, for economic, environmental and health reasons. In future works, a more comprehensive study of the machined parts with the conventional cutting fluid versus the machined parts with the vegetable oils could be analyzed, with a focus on oxidation.

## Figures and Tables

**Table 1:** Machining tests groups.

Test Designation	Lubrication Condition
Dry	Dry
BM50	Balis MAFCOOL 51 with flow rate 50 ± 5 mL/h
BM100	Balis MAFCOOL 51 with flow rate 100 ± 5 mL/h
VO50	Vegetable oil with flow rate 50 ± 5 mL/h
VO100	Vegetable oil with flow rate 100 ± 5 mL/h

**Table 2:** Taguchi array for the tests.

Test Number	A	B	C
**1**	1	1	1
**2**	1	2	2
**3**	1	3	3
**4**	2	1	2
**5**	2	2	3
**6**	2	3	1
**7**	3	1	3
**8**	3	2	1
**9**	3	3	2

**Table 3:** Levels of cutting parameters.

Parameter	Level 1	Level 2	Level 3
Spindle speed [rpm]	195	285	500
Feed rate [mm/min]	20	63	185
Axial depth [mm]	0.3	0.6	1

**Table 4:** Experimental results of average of Ra measurements and chip temperatures.

Test Number	Dry		BMAF 51-flow rate 50±5 mL/h		BMAF 51-flow rate 100±5 mL/h		Vegetable oil-flow rate 50±5 mL/h		Vegetable oil-flow rate 100±5 mL/h	
	T^1^ [°C]	R_a_^2^ [µm]	T^1^ [°C]	R_a_^2^ [µm]	T^1^ [°C]	R_a_^2^ [µm]	T^1^ [°C]	R_a_^2^ [µm]	T^1^ [°C]	R_a_^2^ [µm]
**1**	67.81	0.82	59.84	0.76	45.19	0.72	58.00	0.79	52.71	0.78
**2**	94.48	2.85	82.34	2.78	77.86	2.75	82.46	3.04	77.68	2.66
**3**	105.70	3.93	101.38	3.47	98.61	3.45	97.54	3.71	93.15	3.75
**4**	101.62	0.70	88.09	0.64	78.20	0.61	91.47	0.66	78.32	0.66
**5**	119.69	1.79	112.52	1.7	100.16	1.70	112.15	1.87	100.94	1.82
**6**	80.71	3.38	68.94	3.06	65.03	2.96	66.7	3.21	60.86	3.24
**7**	121.57	0.62	115.54	0.60	101.7	0.51	106.3	0.64	101.05	0.63
**8**	104.68	0.93	90.91	0.84	86.44	0.83	101.03	0.85	92.31	0.83
**9**	115.41	3.47	103.43	3.03	93.85	3.19	104.39	3.59	101.15	3.56
**Average**	101.29	2.05	91.44	1.87	83.00	1.86	91.11	2.04	84.24	1.99

^1^ temperature; ^2^ arithmetic average of roughness profile.

**Table 5:** Average surface roughness of the workpiece, average chip temperature value, and S/Ns ratio on the dry machining tests.

Test Number	$n^{3}$ [rpm]	${V_{f}}^{4}$ [nm/min]	${ae}^{5}$ [mm]	Average of R_a_ [μm]	S/Ns of R_a_ [dB]	Average Temperature [°C]	S/Ns of Temperature [dB]
**Dry1**	195	20	0.3	0.82	1.09	67.81	−36.79
**Dry2**	195	63	0.6	2.85	−9.97	94.48	−39.77
**Dry3**	195	185	1	3.93	−12.50	105.70	−40.83
**Dry4**	285	20	0.6	0.70	2.76	101.62	−40.32
**Dry5**	285	63	1	1.79	−5.37	119.69	−41.82
**Dry6**	285	185	0.3	3.38	−11.58	80.71	−38.31
**Dry7**	500	20	1	0.62	3.99	121.57	−42.04
**Dry8**	500	63	0.3	0.93	0.17	104.68	−40.72
**Dry9**	500	185	0.6	3.47	−11.66	115.41	−41.48

^3^ spindle speed; ^4^ feed rate; ^5^ axial depth

**Table 6:** S/Ns for each parameter in the dry machining tests.

PARAMETER		S/NS OF R_A_ [DB]	S/NS OF TEMPERATURE [DB]
**A1**	n = 195 rpm	−7.12	−39.13
**A2**	n = 285 rpm	−4.73	−40.15
**A3**	n = 500 rpm	−2.49	−41.41
**B1**	$V_{f}\;$= 20 mm/min	2.62	−39.72
**B2**	$V_{f}\;$= 63 mm/min	−5.05	−40.77
**B3**	$V_{f}$ = 185 mm/min	−11.92	−40.21
**C1**	ae = 0.3 mm	−3.43	−38.61
**C2**	ae = 0.6 mm	−6.29	−40.52
**C3**	ae = 1 mm	−4.62	−41.57

**Table 7:** Average surface roughness, average chip temperature, and S/Ns ratio with Balis Mafcool 51 (50 mL/h).

Test Number	$n^{3}$ [rpm]	${V_{f}}^{4}$ [nm/min]	${ae}^{5}$ [mm]	Average of R_a_ [μm]	S/Ns of R_a_ [dB]	Average Temperature [°C]	S/Ns of Temperature [dB]
**BM50_1**	195	20	0.3	0.76	1.78	59.84	−35.73
**BM50_2**	195	63	0.6	2.78	−9.50	82.34	−38.49
**BM50_3**	195	185	1	3.47	−12.24	101.38	−40.37
**BM50_4**	285	20	0.6	0.64	3.24	88.09	−39.05
**BM50_5**	285	63	1	1.7	−5.37	112.52	−41.21
**BM50_6**	285	185	0.3	3.06	−10.66	68.94	−36.95
**BM50_7**	500	20	1	0.60	3.89	115.54	−41.48
**BM50_8**	500	63	0.3	0.84	1.02	90.91	−39.46
**BM50_9**	500	185	0.6	3.03	−10.5	103.43	−40.51

**Table 8:** Average surface roughness, average chip temperatures, and S/Ns ratio with Balis Mafcool 51 (100 mL/h).

Test Number	$n^{3}$ [rpm]	${V_{f}}^{4}$ [nm/min]	${ae}^{5}$ [mm]	Average of R_a_ [μm]	S/Ns of R_a_ [dB]	Average Temperature [°C]	S/Ns of Temperature [dB]
**BM100_1**	195	20	0.3	0.72	2.29	45.19	−33.16
**BM100_2**	195	63	0.6	2.75	−9.53	77.86	−37.98
**BM100_3**	195	185	1	3.45	−12.25	98.61	−40.17
**BM100_4**	285	20	0.6	0.61	3.81	78.20	−38.25
**BM100_5**	285	63	1	1.70	−5.50	100.16	−40.44
**BM100_6**	285	185	0.3	2.96	−10.46	65.03	−36.57
**BM100_7**	500	20	1	0.51	4.99	101.70	−40.60
**BM100_8**	500	63	0.3	0.83	1.23	86.44	−39.12
**BM100_9**	500	185	0.6	3.19	−10.75	93.85	−39.82

**Table 9:** Average surface roughness, average chip temperature values, and S/Ns ratios with vegetable oil (50 mL/h).

Test Number	$n^{3}$ [rpm]	${V_{f}}^{4}$ [nm/min]	${ae}^{5}$ [mm]	Average of R_a_ [μm]	S/Ns of R_a_ [dB]	Average Temperature [°C]	S/Ns of Temperature [dB]
**VO50_1**	195	20	0.3	0.79	1.56	58.00	−35.44
**VO50_2**	195	63	0.6	3.04	−10.47	82.46	−38.58
**VO50_3**	195	185	1	3.71	−12.88	97.54	−40.18
**VO50_4**	285	20	0.6	0.66	2.93	91.47	−39.41
**VO50_5**	285	63	1	1.87	−6.02	112.15	−41.13
**VO50_6**	285	185	0.3	3.21	−10.90	66.7	−36.70
**VO50_7**	500	20	1	0.64	3.29	106.03	−40.91
**VO50_8**	500	63	0.3	0.85	0.89	101.03	−40.39
**VO50_9**	500	185	0.6	3.59	−11.91	104.39	−40.57

**Table 10:** Average surface roughness, average machining temperature, and S/Ns ratios with vegetable oil (100 mL/h).

Test Number	$n^{3}$ [rpm]	${V_{f}}^{4}$ [nm/min]	${ae}^{5}$ [mm]	Average of R_a_ [μm]	S/Ns of R_a_ [dB]	Average Temperature [°C]	S/Ns of Temperature [dB]
**VO100_1**	195	20	0.3	0.78	1.72	52.71	−34.60
**VO100_2**	195	63	0.6	2.66	−9.64	77.68	−38.03
**VO100_3**	195	185	1	3.75	−12.66	93.15	−39.83
**VO100_4**	285	20	0.6	0.66	3.55	78.32	−38.13
**VO100_5**	285	63	1	1.82	−6.02	100.94	−40.30
**VO100_6**	285	185	0.3	3.24	−11.20	60.86	−35.86
**VO100_7**	500	20	1	0.63	3.39	101.05	−40.69
**VO100_8**	500	63	0.3	0.83	1.18	92.31	−39.61
**VO100_9**	500	185	0.6	3.56	−11.87	101.15	−40.37

**Table 11:** ANOVA analysis of chip temperatures and surface roughness of the workpiece after machining tests without lubrification (dry).

Temperature						
	Df^6^	Sq^7^	Md^8^	F Value^9^	*p* Value^10^	Contribution [%]
n	2	7.8523	3.9262	11.41	0.081	33.10%
$V_{f}$	2	1.6615	0.8307	2.41	0.293	7.00%
ae	2	13.5176	6.7588	19.64	0.048	56.99%
**Error**	2	0.6883	0.3442			2.90%
**Total**	8	23.7197				100.00%
**Surface Roughness**						
	**Df^6^**	**Sq^7^**	**Md^8^**	**F Value^9^**	***p* Value^10^**	**Contribution [%]**
n	2	32.14	16.068	2.7	0.271	8.60%
$V_{f}$	2	317.53	158.765	26.64	0.036	84.92%
ae	2	12.33	6.163	1.03	0.492	3.30%
**Error**	2	11.92	5.96			3.19%
**Total**	8	373.91				100.00%

^6^ degrees of freedom; ^7^ sum of squares; ^8^ mean of squares; ^9^ variations between sample means; ^10^ variations within the sample

**Table 12:** ANOVA analysis for chip temperatures and surface roughness of the workpiece after machining tests with Balis Mafcool 51 (50 mL/h).

Temperature						
	Df	Sq	Md	F Value	*p* Value	Contribution [%]
n	2	8.0047	4.0023	11.76	0.078	26.65
$V_{f}$	2	1.4011	0.7005	2.06	0.327	4.67
ae	2	19.9465	9.9733	29.31	0.048	66.42
**Error**	2	0.6805	0.3402			2.27
**Total**	8	30.0327				100.00
**Surface Roughness**						
	**Df**	**Sq**	**Md**	**F Value**	***p* Value**	**Contribution [%]**
n	2	34.23	17.116	2.75	0.266	9.5
$V_{f}$	2	299.97	149.986	24.14	0.04	83.22
ae	2	13.83	6.916	1.11	0.473	3.84
**Error**	2	12.43	6.213			3.45
**Total**	8	360.46				100.00

**Table 13:** ANOVA analysis for chip temperatures and surface roughness of the workpiece after machining tests with Balis Mafcool 51 (100 mL/h).

Temperature						
	**Df**	Sq	Md	F Value	*p* Value	Contribution [%]
n	2	11.300	5.650	3.97	0.201	24.78
$V_{f}$	2	5.769	2.884	2.03	0.330	12.65
ae	2	25.693	12.846	9.02	0.100	56.33
**Error**	2	2.847	1.423			6.24
**Total**	8	45.608				100.00
**Surface Roughness**						
	**Df**	**Sq**	**Md**	**F Value**	***p* Value**	**Contribution [%]**
n	2	37.32	18.662	3.13	0.242	9.39
$V_{f}$	2	332.84	166.419	27.87	0.035	83.22
ae	2	13.83	6.916	1.11	0.436	3.88
**Error**	2	11.94	5.971			3.00
**Total**	8	397.55				100.00

**Table 14:** ANOVA analysis for chip temperatures and surface roughness of the workpiece after machining tests with vegetable oil (50 mL/h).

Temperature						
	Df^6^	Sq	Md	F Value	*p* Value	Contribution [%]
n	2	9.933	4.967	3.84	0.207	31.30
$V_{f}$	2	3.197	1.598	1.23	0.448	10.07
ae	2	16.015	8.008	6.18	0.139	50.46
**Error**	2	2.590	1.295			8.16
**Total**	8	31.735				100.00
**Surface Roughness**						
	**Df**	**Sq**	**Md**	**F Value**	***p* Value**	**Contribution [%]**
n	2	33.11	16.554	2.16	0.317	8.59
$V_{f}$	2	316.06	158.032	20.61	0.046	80.02
ae	2	20.82	10.411	1.36	0.424	5.40
**Error**	2	15.34	7.668			3.98
**Total**	8	385.33				100.00

**Table 15:** ANOVA analysis for chip temperatures and surface roughness of the workpiece after machining tests with vegetable oil (100 mL/h).

Temperature						
	Df^6^	Sq	Md	F Value	*p* Value	Contribution [%]
n	2	12.369	6.1847	7.95	0.116	33.46
$V_{f}$	2	3.434	1.7169	2.11	0.322	9.29
ae	2	19.538	9.7191	11.99	0.077	52.85
**Error**	2	1.630	0.8149			4.41
**Total**	8	36.971				100.00
**Surface Roughness**						
	**Df^6^**	**Sq**	**Md**	**F Value**	***p* Value**	**Contribution [%]**
n	2	29.43	14.713	1.63	0.377	7.49
$V_{f}$	2	329.27	164.637	18.49	0.051	83.75
ae	2	16.63	8.317	0.93	0.517	4.23
**Error**	2	17.81	8.904			4.53
**Total**	8	393.14				100.00

**Table 16:** Wear results for the alumina ball.

Test	Specific Wear Rate (K) [mm^3^/Nm]
Dry	1.98 × 10^−05^
Distilled water	1.72 × 10^−06^
Vegetable oil 1-1st	---
Vegetable oil 1-2nd	---
Vegetable oil 2-1st	---
Vegetable oil 2-2nd	---
Balis MAFCOOL 51	---
